# Supplementary material for: Enhancing Variant Prioritization in VarFish through On-Premise Computational Facial Analysis
Source: Genes (Basel). 2024 Mar 17;15(3):370. doi: 10.3390/genes15030370 (PMC10969976; doi:10.3390/genes15030370)
Supplement: Supplementary file 1 [file genes-15-00370-s001.zip › genes-2885475-supplementary.pdf]

Enhancing Variant Prioritization in VarFish through On-Premise Computational Facial Analysis

Supplementary Material

Meghna Ahuja Bhasin [1], Alexej Knaus [1], Pietro Incardona [1,2], Alexander Schmid [1], Manuel Holtgrewe [3], Miriam Elbracht [4], Peter M. Krawitz [1] and Tzung-Chien Hsieh [1,*]

1 Institute for Genomic Statistics and Bioinformatics, University Hospital Bonn, Rheinische Frie-drich-Wilhelms-Universität Bonn, Bonn, Germany;
2 Core Unit for Bioinformatics Data Analysis, Medical Faculty, University of Bonn, Bonn, Germany
3 CUBI – Core Unit Bioinformatics, Berlin Institute of Health, Berlin 10117, Germany;
4 Institute for Human Genetics and Genomic Medicine, Medical Faculty, RWTH Aachen University, Aachen, Germany
* Correspondence: thsieh@uni-bonn.de

Instruction of starting each service in VarFish

1. To launch VarFish server, first clone the git repository from https://github.com/ahujameg/varfish-server.git
   Then set the environment and start VarFish:
   ```
   pipenv shell
   pipenv sync
   python manage.py runserver
   ```
   Start celery in another terminal:
   ```
   make celery
   ```
   Start Vueapp in another terminal (first make sure NodeJS and NPM are installed):
   ```
   cd <path to varfish-server>/varfish/vueapp
   npm run build
   npm run serve
   ```
2. To start CADD server, follow the VarFish documentation: https://varfish-server.readthedocs.io/en/latest/admin_extras.html?highlight=CADD#install-scoring-with-cadd

   Example request payload:
   {

"genome_build": "GRCh37",
"cadd_release": "v1.6",
"variant": [
"1-25893242-TG-CA",
"4-100544433-T-A",
"8-19824667-CAC-TAA",
…
]
}

Example response:

{
"result": "OK",
"status": "finished",
"scores": {
"1-25893242-TG-CA": [
-0.079176,
1.612
],
"4-100544433-T-A": [
0.212352,
6.022
],
"8-19824667-CAC-TAA": [
0.388051,
8.268
],

},
"args": {
"genome_build": "GRCh37",
"cadd_release": "v1.6",
"variants": [
"1-25893242-TG-CA",
"4-100544433-T-A",
"8-19824667-CAC-TAA"
]
},
"info": {
"cadd_rest_api_version": 0.1
}
}

3. The implementation of CADA is available at https://github.com/Chengyao-Peng/CADA and it's web service can be accessed from here https://cada.gene-talk.de/api/process.

   Example request payload:

```
{
    ["HP:0001167", "HP:000118"]
}
```

Example response:

```
{[
    {
        "geneId": 80155,
        "geneSymbol": "NAA15",
        "score": 0.255019928018252
    },
    {
        "geneId": 5058,
        "geneSymbol": "PAK1",
        "score": 0.2413779695828755
    },
    …
}]
```

4. To run the GestaltMatcher service, follow the steps mentioned here: https://github.com/igsb/GestaltMatcher-Arc/tree/service#gestaltmatcher-rest-api

5. To run PEDIA Middleware service, clone the repository at https://github.com/igsb/pedia-middleware Set the environment:

```
pipenv shell
pipenv sync
```

Start the application server on a different port than Varfish, for example on port 7000:

```
python manage.py runserver 7000
```

6. To run PEDIA classifier web service, clone the repository at https://github.com/PEDIA-Charite/classifier
Build docker image:

```
docker build -t pedia-api .
```

Run and listen the request in localhost:9000

```
docker run -p 9000:9000 pedia-api
```

Example request payload:

```
[
    {
        "gene_name": "TTN",
        "gene_id": 7273,
        "cada_score": 0.15950778339590344,
        "cadd_score": 23.9,
        "gestalt_score": 0.993,
        "label": "False"
    },
    {
```

"gene_name": "CDC42BPA",
"gene_id": 8476,
"cada_score": 0.16774456841605048,
"cadd_score": 18.5,
"gestalt_score": 0.991,
"label": "True"
},
…
]

Example response:

[
{
"gene_name": "CDC42BPA",
"gene_id": 8476,
"pedia_score": 1.6125531907965023,
"cadd_score": 18.5,
"gestalt_score": 0.991,
"cada_score": 0.16774456841605048,
"label": 1
},
{
"gene_name": "TTN",
"gene_id": 7273,
"pedia_score": 1.8549785017694944,
"cadd_score": 23.9,
"gestalt_score": 0.993,
"cada_score": 0.15950778339590344,
"label": 0
},
…
]
